# The role of indocyanine green in fluorescence-guided pancreatic surgery: a comprehensive review

**DOI:** 10.1097/JS9.0000000000002311

**Published:** 2025-02-27

**Authors:** Andrea Tufo, Anna Caterina Milanetto, Roberto Valente, Enrico Spalice, Loredana Sodano, Claudio Pasquali, Maria Chiara Scandavini, Alessandro Coppola

**Affiliations:** a UOC Chirurgia Generale, Ospedale del Mare, Napoli, Italy; b Department of Surgery, Oncology and Gastroenterology, University of Padova, Padova, Italy; c Department of Diagnostic and Intervention, Surgery, Umeå University, Umeå, Sweden; d Division of Surgical Oncology, Department of Surgery, University of Colorado School of Medicine, Aurora, Colorado, USA; e Department of Surgery, Sapienza University of Rome, Rome, Italy

**Keywords:** indocyanine green, mininvasive surgery, pancreas surgery, pancreatic cancer, precision surgery

## Abstract

Pancreatic surgery is a complex and challenging field, with patients facing a high risk of postoperative complications. In recent years, indocyanine green (ICG) has gained prominence as a valuable tool used in various aspects of pancreatic surgery. ICG is a fluorescent dye that offers real-time imaging capabilities that enhance the surgeon’s ability to accurately localize tumors and critical anatomical structures, thereby improving surgical precision and potentially reducing operative time and complications. One of the most significant advantages of ICG is its ability to provide enhanced visualization of the biliary tract and vascular structures, which is particularly beneficial in complex pancreatic resections, in which the anatomy can be highly variable and challenging to navigate. Furthermore, ICG can be instrumental in ensuring the adequate perfusion of anastomoses, thereby reducing the risk of postoperative leaks and associated morbidity. This comprehensive review aims to provide an in-depth analysis of the current applications, advantages, and limitations of ICG in pancreatic surgery.

**HIGHLIGHTS**

- Despite the considerable advances that have been made in perioperative and clinical care, surgical resection remains a challenge even in experienced hands, and it is associated with a high rate of postoperative complications and deaths.
- Indocyanine green (ICG) has gained prominence as a valuable tool used in various aspects of pancreatic surgery.
- ICG applications include the intraoperative identification of pancreatic tumors and metastases, node navigation surgery, lymph node mapping during lymphadenectomy, the assessment of the vascular and biliary anatomy, and the evaluation of anastomotic perfusion.
- The role of ICG in pancreatic surgery is far from defined; as reported in this narrative review, its applications and benefits can be manifold

## Introduction

Surgical resection plays a central role in the treatment of both benign and malignant pancreatic tumors. Despite the considerable advances that have been made in perioperative and clinical care, surgical resection remains a challenge even in experienced hands, and it is associated with a high rate of postoperative complications and deaths[1].

Over the last two decades, researchers have increasingly focused on developing novel approaches to enhance the precision of surgical resection and improve oncological outcomes while minimizing postoperative complications. Notably, minimally invasive surgical techniques – including laparoscopy and, more recently, robotic surgery – have emerged as pivotal advancements in this pursuit. These methods have undergone rigorous validation and have been embraced by specialized hepato-biliary-pancreatic (HPB) centers[2].

Robotic surgery, in particular, has played an important role in transitioning from a practice that is limited to a select group of highly skilled surgeons to a more universally reproducible approach that is aided by the integration of intraoperative navigation tools. Among the array of emerging technologies, near-infrared (NIR) fluorescence imaging using ICG has gained widespread adoption. ICG is a NIR fluorescent dye that is administered intravenously, binds to plasma proteins, and disperses within the intravascular space. ICG exhibits a peak spectral absorption range between 600 and 800 nm, and it has a relatively short half-life of 3–4 minutes before being excreted into the bile without undergoing metabolic changes, typically within 15–20 minutes. Its wavelengths permit tissue penetration of up to 1 cm in depth, enabling visualization of the dye within tissues up to 8 mm in thickness. Furthermore, ICG’s affinity for lymphatic tissues, which are high in protein content, facilitates its accumulation in lymph nodes, thereby facilitating the precise visualization of nodal stations. This visualization is made possible through the use of radiation-free fluorescence imaging systems, which employ both infrared and LED light sources. These systems incorporate filters that seamlessly transition to fluorescence mode, allowing real-time display on monitors and enhancing surgical guidance without the need for ionizing radiation[3].

ICG was approved for clinical use in 1956 and was initially utilized for performing angiographies, assessing hepatic function, determining cardiac output and evaluating liver and gastric blood flow[4].

With the advent of fluorescence-guided surgery, the scope of ICG’s application has broadened, and it is now used routinely in breast, lung, liver, and colorectal surgeries for cancer identification, lymphatic system identification, anatomy visualization, and perfusion assessment[5].

This study aims to describe the applications of fluorescence imaging in pancreatic surgery to consolidate its role in advancing surgical practice and patient care.

## Methods

A search of the PubMed database for articles published up to October 2023 was carried out. Different combinations of the following search terms were used: indocyanine green, pancreas, pancreas surgery, pancreatic surgery, and nodal involvement. Only articles published in English with available full text were considered, regardless of the article type (original articles, review, etc.) or date of publication. References cited in the selected articles were also considered as bibliographic sources.

## Results

NIR imaging using ICG has demonstrated promising utility across several aspects of pancreatic surgery. Originally used for visualizing the biliary system, this imaging now serves as a method for marking areas before surgery to precisely locate tumors, thus helping to ensure complete tumor removal and assisting in identifying lymph nodes. Furthermore, this technique proves valuable in pinpointing anatomical structures, especially the complex network of vessels around the pancreas. Its benefits extend to evaluating blood flow after vascular reconstruction. Although it is increasingly popular in minimally invasive pancreatic surgery, near-infrared imaging with ICG allows for multiple real-time evaluations during surgery and so proves equally useful in traditional open procedures. Fluorescence imaging has three major applications during surgery:

- The visualization and staging of cancers
- The identification of anatomic structures
- The assessment of organ perfusion

### Cancer visualization and staging

In pancreatic surgery, preserving as much parenchyma as possible is essential to minimize the risk of endocrine and exocrine insufficiency, especially in cases of small, benign, or low-grade malignant tumors such as the neuroendocrine tumors (PanNets). Furthermore, the resection of pancreatic malignancies is often complicated by high rates of local and distant recurrence due to positive margins and unrecognized metastases. Enhancing tumors visualization could lead to improved outcomes. In this context, fluorescence-guided surgery has emerged as an innovative intraoperative technique that aids surgeons in those critical tasks.

#### Pancreatic ductal adenocarcinoma

In conventional pancreatic surgery for pancreatic ductal adenocarcinoma (PDAC), the processes of tumor localization and the assessment of disease extension traditionally rely on visual inspection together with palpation. However, this approach carries the risk of incomplete resection or the unnecessary removal of healthy tissue. Image-guided surgery using ultrasonography (US) offers a solution to this problem by enabling the real-time, intraoperative visual identification of pancreatic tumors with enhanced discrimination between malignant and normal tissues. To confirm the absence of tumor at the resection margin intraoperative frozen-section analysis (IFSC) is routinely used during pancreatic surgery.

However, both US and IFSC have limitations when used to assess pancreatic tumor extension intraoperatively.

IFSC is time-consuming and sometimes lacks precision. While is effective in preventing negative margins, it has a predictive negative value of only 50% when evaluating pancreatic lesions. This limitation can lead to inadequate histological diagnoses and affect the determination of resectability.[6]

In 2011, Hutteman *et al* conducted a clinical trial evaluating the utility of NIR imaging with ICG for the intraoperative detection of pancreatic tumors and the assessment of the resection margin.[7] They performed NIR imaging immediately following the intraoperative administration of 5–10 mg of ICG in patients undergoing pancreaticoduodenectomy. Regardless of the early enthusiasm on this technique, the authors concluded that ICG did not provide effective tumor delineation when a low dose of ICG was administered during surgery. However, when a higher dose administered 24 hours before surgery (the second-window ICG technique), ICG accumulated in tumors because of the enhanced permeability and retention (EPR) effect. The EPR effect was initially described by Matsumura *et al* and posits that tumor angiogenesis results in the formation of excessively permeable capillaries[8]. When ICG is injected into the bloodstream, it has no known metabolites and binds primarily to lipid or lipoprotein complexes, with the bound protein acting as a macromolecule. Due to the presence of defective, leaky capillaries in tumors, these macromolecules become trapped within the tumors, while most ICG molecules are cleared from normal tissues. Consequently, during the extended imaging window, the high concentration of ICG in tumors and the negligible amount in normal tissues creates a significant difference in fluorescence signals between cancerous and noncancerous tissues. This results in improved image quality in ICG-NIRF-I imaging during the long imaging window compared to the short one. Over time, ICG molecules are gradually extracted by the liver into bile, leading to a decrease in ICG concentration – and consequently, a reduction in fluorescence signals – in the tumor during the long imaging window. As a result, the tumor-to-background ratio (TBR) of ICG-NIRF-II diminishes in the long imaging window compared to the short imaging window.[9]

Most existing studies using ICG have focused on imaging within the first near-infrared window (NIR-I, 700–1000 nm). In contrast, imaging in the second near-infrared window (NIR-II, 1000–1700 nm) could offers greater tissue penetration and improved image quality due to reduced autofluorescence and photon scattering, particularly when using ICG.

In 2019, Newton *et al* conducted a prospective open-label clinical trial demonstrating that intraoperative NIR imaging with second-window ICG (5 mg/kg administered 24 hours prior to surgery) effectively identified invasive pancreatic malignancies, including PDAC. With this imaging approach among 11 patients with PDAC, 91.7% (11 out of 12) displayed fluorescence, with a mean TBR of 4.62 ± 2.95. Notably, one patient with a nonfluorescent PDAC tumor (TBR 1.25) had undergone preoperative chemoradiotherapy, resulting in only 10% remaining viable tumor. In addition, fluorescence observed at the resection margin correlated with final pathological outcomes in 12 of 13 cases. The positive predictive value for fluorescence at the pancreatic neck margin was 83.3% (5 out of 6), while the negative predictive value reached 100% (7 out of 7). ICG fluorescence accurately predicted positive margins in all four relevant cases, revealing extensive disease that was previously underestimated by preoperative imaging in three instances. This underscores a significant advantage of NIR imaging: these patients might have avoided extensive resections with positive margins and could have been directed toward neoadjuvant therapy or definitive chemotherapy/chemoradiotherapy.[10]

Another significant challenge during pancreaticoduodenectomy (PD) has always been the assessment of the retroperitoneal margin due to the close anatomical proximity of the uncinate process of the pancreas to the superior mesenteric artery (SMA), which is intertwined with the nerve plexus and surrounding soft tissues. Whereas in traditional open surgeries palpation of the SMA may aid in defining the dissection line to secure the margin of the uncinate process, the ability to do this is limited in the laparoscopic era. In standard white light, distinguishing the pancreatic tissue of the uncinate process from the surrounding soft tissues, including the nerve plexus around the SMA, proves challenging[11]. Rho *et al* conducted a study in which they employed an ICG-enhanced approach in ten patients who were undergoing laparoscopic PD for periampullary tumors. ICG was intravenously injected (5 mg/2 cm^3^) during the dissection of the uncinate process, which offered real-time visual feedback and enabled the clear visualization of the pancreatic tissue, which would otherwise be indistinguishable from the nerve plexus around the SMA[12].

**Figure 1.**
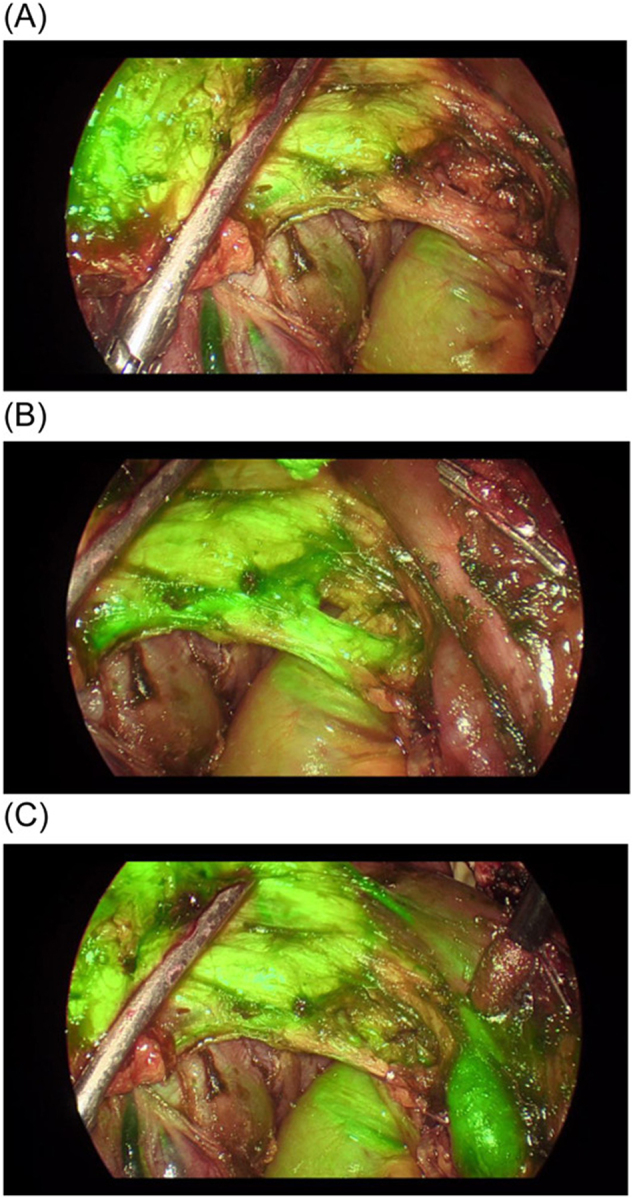
Pancreatic uncinate process and adjacent vessels during different timing after ICG bolus. (A) Before IV ICG bolus, with continuous infusion. (B) Arterial phase, 10 s after bolus. Notice the increased fluorescence of a superior mesenteric artery branch to the pancreas. (C) Venous phase, 40 s after bolus. Superior mesenteric vein is hyperfluorescent. ICG, indocyanine green; IV, intravenous. Courtesy of Abun *et al*.[12]

To enhance visualization of the pancreas while minimizing interference from surrounding tissues, Asbun *et al* suggested using continuous ICG infusion. The injection is generally administered just after the laparotomy for distal pancreatic tumor, and just after bile duct resection for head of pancreas procedures, and the ICG can be infused at the rate of 0.4 per minute. They showed the utility of this technique in a video for a difficult uncinate process dissection during pancreaticoduodenectomy in a patient with a history of pancreatitis[13] (Fig. 1).

Currently, there are no optimal methods for assessing the response of pancreatic cancer to neoadjuvant therapy. Imaging techniques often fail to correlate with the treatment response or the feasibility of achieving an R0 or R1 resection. Tumor markers can be informative but should not be used in isolation to make decisions regarding high-risk resections. In the study by Newton *et al*, four patients underwent neoadjuvant therapy. Notably, fluorescence imaging aligned with pathological findings in all cases. The ability to identify poor treatment responses prior to resection may influence the decision to proceed with surgery. Therefore, integrating NIR imaging into intraoperative decision-making could be beneficial for patients with borderline resectable or locally advanced PDAC following neoadjuvant therapy.[10]

Despite those encouraging results, there are limitations associated with the use of ICG in PDAC. Since ICG is cleared hepatically, it results in increased background fluorescence in the pancreatic head region that can obscure imaging during pancreaticoduodenectomy. In contrast, background fluorescence does not pose a challenge during distal pancreatectomy, facilitating more precise tumor localization in that context.[10]

Moreover, ICG lacks specificity for pancreatic cancer. A recent Delphi consensus survey underscored this limitation and recommended moving away from the use of ICG for visualizing pancreatic tumors. Instead, it advocated for the continued development and translation of tumor-targeted probes[14].

#### Pancreatic neuroendocrine tumours

Most researches on pancreatic cancer have predominantly concentrated on patients with PDAC, that account for the majority of pancreatic tumors. Only 5 % of pancreatic neoplasms is represented by pancreatic neuroendocrine tumors. Accurately identifying small PanNETs suitable for minimally invasive, parenchyma-sparing resections, such as enucleation, can be quite challenging. Particularly during minimally invasive procedures, the overall visibility of the pancreas and also tactile sensations are restricted.^[^15,16^]^

Conventional preoperative imaging routinely utilized for the detection and staging of PanNETs, such as computed tomography (CT) scanning, magnetic resonance imaging (MRI), and 68 Ga-Positron Emission Tomography/CT, exhibit a high detection rate for larger tumors, but they may yield suboptimal results for smaller tumors and insulinomas[17].

In such cases, fluorescence-guided surgery (FGS) may offer significant advantages. Shirata *et al* demonstrated that fluorescence intensity values were notably higher in NETs compared with the surrounding parenchyma (with 1.99 times higher fluorescence intensity), which was attributed to the richer vascularity of PanNETs[15]. In a study by Paiella *et al* on a series of 10 patients with PanNETs (both functioning and nonfunctioning) with a mean maximum tumor dimension of 2.4 cm (range, 1–4 cm), all of them were identified using NIR imaging after the IV bolus administration of ICG, with a peak visualization achieved 20 minutes after the last bolus[16].

Unlike adenocarcinoma, PanNETs are easily identified with ICG both in the first (700–1000 nm) and second near-infrared windows (1000–1700 nm) as reported by Li *et al*. In their study involving nine patients with PanNETs, tumor tissues exhibited brighter fluorescence compared with normal tissues, whereas the margins displayed a similar fluorescence intensity to those of normal pancreatic tissues, indicating negative margins as was confirmed by histopathology[18].

Kou *et al* analysed the experiences reported by other authors regarding the use of NIR employing ICG to localize PanNETs^[^15,16,19-21^]^. Their findings indicated a tumor identification rate of 100% irrespective of the tumor’s solid or cystic nature or its functional status. In a recent review of Rompianesi *et al* the intraoperative visualization of 21 out of 24 NETs was achieved through ICG fluorescence, yielding a positive predictive value (PPV) of 0.913 (95% CI 0.711–0.978). The sensitivity of ICG fluorescence in detecting pancreatic NETs was determined to be 1.000 (95% CI 0.930–1), although a specific specificity calculation was not feasible[22] (Table 1).

**Table 1:** Identification of the bile duct

Case/Study	Patient Details	Procedure	Use of ICG	Findings	Outcome
Ishizawa T. *et al*[28]	52 patients undergoing laparoscopic cholecystectomy	Laparoscopic cholecystectomy	2.5 mg ICG injected 30 min before surgery or post-intubation	Fluorescent cholangiography delineated cystic duct in all patients, cystic duct-common hepatic duct junction in 50 patients	Enabled real-time identification of biliary anatomy, potentially replacing radiographic cholangiography
Hutteman, M. *et al*[7]	8 patients undergoing pancreaticoduodenectomy	Pancreaticoduodenectomy	5 or 10 mg ICG injected intravenously	No clear tumor-to-pancreas contrast except in 1 patient; common bile duct clearly visualized within 10 min, maximal contrast 30-90 min post-injection	Useful for visualizing common bile duct and biliary anastomoses, not for tumor demarcation
Cao J. *et al*[34]	12 patients (10 women, 2 men; avg. age 37.3 years)	Laparoscopic Duodenum-preserving total pancreatic head resection (LDPPHRt)	No specific ICG use	Average operative time: 272.5 min; average blood loss: 215 ml; 16.7% had pancreatic fistula, 16.7% had biliary fistula	Successful LDPPHRt with good organ preservation; complications managed conservatively
Lu C. *et al*[35]	25 patients	L-DPPHR	Different ICG doses/timing preoperatively; evaluated intra-operatively	Best guidance with 0.5 mg/kg 24 h before operation; ICG decreased bile leakage from 10% to none	Feasible, safe, decreased bile duct injury; no tumor recurrence, good metabolic outcomes
Cai Y. *et al*[36]	24 patients (9 males, 15 females; median age 43 years)	LDPPHR	Real-time ICG fluorescence imaging	Median operative time: 255 min; median blood loss: 200 ml; 45.8% had pancreatic fistula, 4.2% had grade B fistula	Safe, feasible; no grade C pancreatic fistula; useful for preventing bile duct injury

Newton *et al* observed that intraoperative NIR imaging provided valuable information in 18 out of the 20 patients as an adjunct to the processes of intraoperative ultrasound in terms of tumor identification, disease extension, margin assessment, and the prediction of benign disease[10].

ICG has also shown utility in identifying NET lesions in infants. Delgado-Miguel *et al* documented a case of focal congenital hyperinsulinism that was successfully localized during laparoscopy-assisted surgery using ICG-guided NIRF. They administered an intravenous injection of 16 mg ICG (2 mg/kg) and the precise intraoperative localization of the focal lesion enabled the performance of a limited resection.[23]

NIR imaging could offer an advantage in ruling out multifocal PanNETs in the residual parenchyma intraoperatively, as highlighted by Kou *et al*[19] and as reported by Handgraaf *et al* where NIR imaging successfully identified an additional tumor that was not detected by preoperative studies in a case of multiple insulinomas[20].

To guide the resection of insulinomas, Tao *et al* introduced a two-stage ICG injection technique during the laparoscopic enucleation. Initially, they injected 1 mg of ICG into the peripheral vein for tumor localization. Subsequently, ICG (0.025 mg/ml, total volume 1–2 ml) was injected under the pseudo-capsule of the insulinoma for obtaining real-time 3D demarcation, thus complementing intraoperative ultrasound. During enucleation, the ICG fluorescence imaging showed the 3D resection plane, and the postenucleation assessment involved evaluating the residual ICG fluorescence on the pancreatic surface^[^16,24^]^.

In fact, the effectiveness of ICG can be significantly enhanced when combined with intraoperative ultrasound. In the study of Newton *et al*, together, these tools facilitate the identification of the tumor’s proximity to the pancreatic duct, aiming to prevent duct injury and the subsequent development of pancreatic fistula.[10]

In addition, the ICG dynamic perfusion could help surgeons to observe any abnormal vascular perfusion in PanNETs and could provide information that was comparable to that from intraoperative real-time angiography.[24]

The utility of ICG in the intraoperative identification of PanNETs may be subject to certain limitations. Variations in ICG uptake within the pancreatic parenchyma pose a challenge, particularly in patients with high levels of pancreatic or peripancreatic fat tissue, in which fluorescence may be diminished. This variability in uptake could limit the effectiveness of the ICG technique, particularly in detecting insulinomas given that patients with insulinomas often have obesity-related fatty pancreatic parenchyma[13].

The optimal timing for intravenous ICG administration appears to be intraoperative owing to the absence of influx and efflux transporters in the pancreatic tissues and lesions[25].

Huang *et al* reported that in their experiences, in particular in patients with inflamed or thickened tissues in obese patients they administered 0.5 mg/kg ICG intravenously 24 h before surgery and subsequently administered an additional 5 mg of ICG intravenously during surgery to improve the quality of intraoperative imaging that is usually very poor in those circumstances[26].

However, further investigations on ICG usage in PanNETs surgery may focus on optimizing the timing and dosage of ICG injection, which may vary depending on each patient’s characteristics[13].

Nonetheless, the constant need to shift visual focus between the laparoscopic surgery system and the intraoperative ultrasound system can be fatiguing and distracting for the surgeon.

#### Liver metastases

ICG fluorescence serves a vital role in the detection of liver micro-metastases, which is an essential task when managing pancreatic cancer due to its significant prognostic implications. As conventional preoperative imaging techniques, such as CT and MRI, often lack the sensitivity required to detect micro-metastases effectively, alternative approaches are needed[11].

In this context, the administration of ICG before commencing a staging laparoscopy emerges as a promising strategy. By using ICG, clinicians may discover liver metastases that conventional imaging modalities might overlook, thus enhancing staging accuracy and guiding treatment decisions^[^20,27^]^.

In a recent study of Handgraaf *et al*, 25 patients received 10 mg of ICG one day before surgery. During the surgical procedure, intraoperative fluorescence imaging and laparoscopic ultrasound of the liver were conducted, and biopsy or resection was performed for suspected metastatic lesions. The results demonstrated the superior accuracy of ICG in detecting liver metastases compared with visual inspection and laparoscopic ultrasound[20].

However, the effectiveness of ICG is constrained by the depth of tumors beneath the liver’s surface due to the limited tissue penetration of near-infrared light. Notably, tumors located more than 8 mm beneath the surface of the liver were not identifiable in the study. Despite this limitation, intraoperative ICG-fluorescent imaging remains invaluable as it aids surgeons in visual inspections, palpation, and the identification of residual cancerous tissues postresection[28].

Yokoyama *et al* applied this procedure in a preliminary study to identify radiographically occult hepatic micro-metastases from PDAC in 49 consecutive patients. Preoperative imaging, intraoperative inspection, and intraoperative US failed to reveal any liver metastases in these patients. ICG was administered intravenously one day before surgery and fluorescing lesions were subsequently identified on the hepatic surface in 13 patients. The lesions that were detected using ICG fluorescence were resected and examined via frozen-section histology; histological analysis confirmed metastatic carcinoma in 17 specimens that were collected from eight patients. It is noteworthy that hepatic micro-metastases originating from pancreatic tumors exhibited fluorescing foci with significantly larger areas in images than that of the actual tumor. This intriguing finding suggests that the observed fluorescence might be indicative of the extent of bile stasis that is induced by the micrometastasis rather than of direct fluorescence from the metastatic cancer cells themselves[29]. These findings were confirmed in a study by Katada *et al* in which all of the micro-metastases were located in the portal triad as portal thromboemboli, and they had invaded extravenous structures causing desmoplasia, local biliary obstruction, and ICG-containing bile stasis[30].

#### Lymph node mapping

Fluorescence imaging has also been performed to assess peripancreatic lymphatic drainage, and it has been suggested for adequate lymph node harvest^[^12,13,31,32^]^.

Hirono *et al* tried to identify the lymphatic drainage pathways from the pancreatic head with guidance from ICG fluorescence imaging in order to plan a lymphadenectomy during PD for PDAC. In their study, several lymphatic drainage routes from the pancreatic head were observed, and the lymphatic drainage was more often (in 85% of cases) directed toward the para-aortic region than to the left side of the SMA (jejunal arteries regions). Therefore, the authors concluded that a lymphadenectomy of the left side of the SMA – including the regions of the first, second, and third jejunal branches – might have similar oncological effects to those of a lymphadenectomy around the paraaortic region. The circumferential clearance of the SMA may not be as beneficial as para-aortic lymph node dissection is for patients with PDAC, although randomized controlled studies are needed to confirm this observation.[32]

Some years later, Matsuki *et al* evaluated the lymphatic flow in the mesopancreas using ICG fluorescence imaging with an intestinal derotation technique to clarify the optimal extent of mesopancreas excision and lymphadenectomy in pancreatoduodenectomy procedures that were performed to treat periampullary tumors. In this study, lymphatic pathways from the pancreatic head reached the SMA via the inferior pancreato-duodenal artery, the first jejunal artery, and finally the paraaortic region. Therefore, the authors concluded that dissecting all areas of lymph nodes that were identified by ICG fluorescence (including the paraaortic region) does not necessarily improve a patient’s prognosis. The optimal lymphadenectomy to perform during pancreatoduodenectomy might thus involve the area comprising the inferior pancreato-duodenal artery and the first jejunal artery, whereas it might be possible to preserve the region of the second jejunal artery and the more distant jejunal arteries[33].

Paiella *et al* used an ICG technique for the intraoperative localization of PanNETs in which they identified two additional lesions that were shown by final histology to be normal lymph nodes[16]. Thus, when using NIR with ICG for lymph node sampling in PanNETs, one should take into account the fact that peripancreatic regional lymph nodes can emit a fluorescence signal even if they are not metastatic[16].

However, the overall risk of false positives when applying NIR in surgical procedures for PanNETs may be reduced either by using pancreatic ultrasound when a NIR-signal is present within the pancreatic parenchyma or by using IFSC in cases with extra-pancreatic NIR-positive lesions.

The authors also speculate that when there is high preoperative suspicion of lymph node involvement, NIR with ICG could allow surgeons to perform frozen-section assessment of regional or distant lymph nodes to ensure that an adequate lymphadenectomy is performed, even if its impact on survival is still debated[16].

### Identification of anatomic structures

#### Bile and pancreatic duct

**Figure 2.**
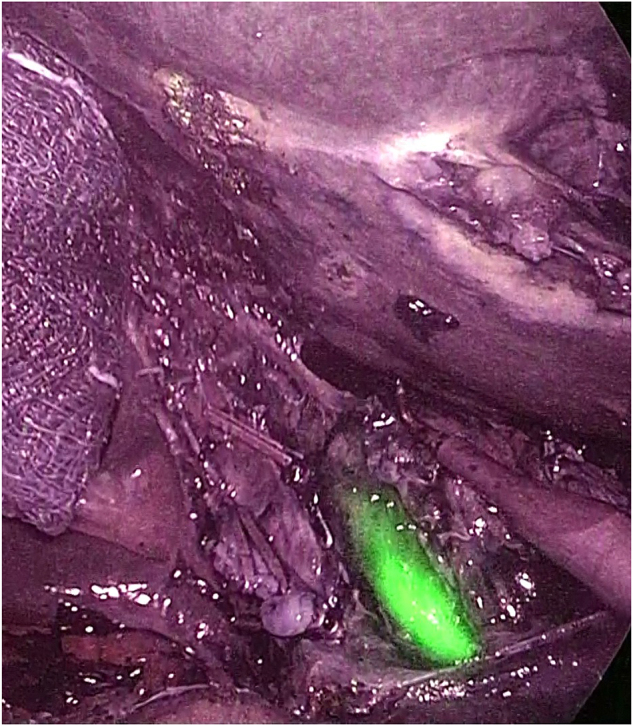
Common bile duct identification. Courtesy of Dr. D. Marchi unpublished picture.

In 2009, Ishizawa *et al* first introduced ICG fluorescence imaging to enhance the real-time visualization of the biliary anatomy during surgery; they were aiming to improve the precision of resection and mitigate the risk of bile ducts injury and bile leaks[28]. Compared with conventional X-ray cholangiography, ICG offers several advantages: it is nonradioactive, cost-effective, and provides high spatial-temporal resolution (Fig. 2).

In pancreatic surgery, ICG holds promise for evaluating the patency of jejunal-biliary anastomosis in patients undergoing PD[7]. Moreover, it serves as a navigational aid akin to a “GPS” around the biliary tree, and it is particularly valuable in the management of complex procedures such as duodenum-preserving pancreatic head resection (DPPHR), where the risk of bile injury and subsequently, bile leak varies between 11.8% and 16.7% due to the blind dissection behind the pancreas during the procedure^[^7,28,34-36^]^. ICG fluorescence imaging might also prove valuable in promptly detecting minor bile duct perforations during surgery, and in revealing the presence of ICG-infused bile in the abdominal cavity[36] (Table 2).

**Table 2:** Pancreatic neuroendocrine tumours (PanNets) detection

Case/Study	Patient Details	Procedure	Use of ICG	Findings	Outcome
Shirata C. *et al*[15]	23 patients undergoing pancreatic resection	Pancreas resection	ICG fluorescence imaging	Neuroendocrine tumors visualized as fluorescence, cystic neoplasms as fluorescence defect	Technique useful in visualizing pancreatic lesions during surgery
Paiella, S. *et al*[16]	6 cirrhotic patients (liver transplantation) and 5 kidney-pancreas transplantation patients	Liver and kidney-pancreas transplantation	ICG fluorescence to assess perfusion	No biliary complications or anastomotic issues reported	Provided important perfusion information, helped in real-time surgical decisions, and reduced risk of complications
Kou H.W. *et al*[19].	29-year-old female with 1.2 cm cystic insulinoma	Resection of pancreatic cystic insulinoma	NIR imaging	Tumor localized easily, safe margin identified, normal pancreatic structure preserved	Pathology confirmed well-differentiated cystic insulinoma; technique was safe and effective
Handgraaf, H. *et al*[20].	25 patients with pancreatic or periampullary cance	Staging laparoscopy (SL) with laparoscopic ultrasonography (LUS) and laparoscopic near-infrared fluorescence imaging (LFI)	10 mg ICG 1-2 days before surgery	Identified liver and peritoneal metastases, high quality LFI in 89% dosed two days prior	Averted futile laparotomy in 3 patients (12%), 2 patients developed metastases within 3 months

A longstanding technical challenge has been determining the optimal timing and dosage of ICG administration to achieve clear visualization of the bile duct anatomy^[^37,38^]^.

When administered intravenously, safety considerations dictate that doses of ICG generally fall between 0.025 and 0.5 mg/kg. Exceeding this range has been associated with adverse reactions such as nausea, fever, and shock[39]. There is not a standard protocol for the timing of injection, and common timing ranges from the day before surgery to just 20 minutes before beginning the dissection; the intraoperative administration of ICG may offer a safer alternative in cases of potential anaphylactic reactions[40].

In their study involving 25 patients undergoing laparoscopic DPPHR (LDPPHR), Lu *et al* employed ICG-guided dissection in 15 patients using a variety of dosages (1, 0.5, and 0.25 mg/kg) and administration timings (IV administration 12 h, 24 h, and 36 h before surgery). Their findings demonstrated that ICG significantly enhanced the detection ability (93.3% vs. 50%, *P* = 0.045) and accuracy of identification (100% vs. 60%, *P* = 0.098) of the CBD compared with the surgery without ICG. The dosage of 0.5 mg/kg administered 24 h before surgery exhibited the highest signal-to-noise ratio. Furthermore, in patients deemed to be at high risk of bile duct injury, an additional intra-biliary dose of 12.5 mg/5 ml was administered to detect suspicious injury sites. The ICG-guided group experienced a lower incidence of bile leakage (0 vs. 10%) and, after a median follow-up of 27 months, the authors observed no refractory cholangitis[35].

Similarly, Huang *et al* reported comparable outcomes in their recent study involving 30 patients undergoing LDPPHR, with ICG guidance utilized in 10 patients. Their results revealed reduced incidence rates of bile leakage (10% vs. 60%) and bile duct injury (0% vs. 13%), in addition to shorter operative times (*P* < 0.05). The authors advocated for the administration of 0.5 mg/kg ICG 24 h before surgery. In cases of obese patients or those with inflamed tissue, the authors recommended an additional intraoperative injection of 5 mg ICG if the initial dose proved to be insufficient for intraoperative imaging. Notably, the benefits of ICG guidance were particularly evident in patients with chronic pancreatitis, in whom distinguishing the boundary between the lesion and the CBD was often challenging with the naked eye[41].

Furthermore, the utility of ICG fluorescence imaging in LDPPHR was amplified in a recent study in which it was integrated into preoperative three-dimensional (3D) models, which facilitated precise anatomical dissection and mitigated the risk of causing inadvertent damage to crucial vessels and the biliary tract[42].

In another study, Ito *et al* demonstrated the utility of ICG fluorescence in visualizing the accessory pancreatic duct during laparoscopic pancreas-preserving duodenectomy (PPD) for second portion duodenal adenocarcinoma[43]. Unlike in PD, in which only the main pancreatic duct is typically exposed, in the pancreatico-jejunostomy of PPD both the main pancreatic duct and the accessory pancreatic duct are visualized. A large-scale multicenter retrospective study emphasized the critical importance of recognizing and appropriately managing aberrant pancreatic ducts to prevent potentially fatal complications[44]. Ito *et al* administered 0.025 mg of ICG via a tube inserted into the main pancreatic duct, consequently revealing a small secondary duct that was successfully closed with sutures and leading to an uneventful recovery for the patient[43]. However, this approach may not be applicable in cases of pancreatic divisum, in which the two ducts are not connected.

### Assessment of tissue perfusion

The evaluation of tissue and organ perfusion via fluorescence imaging represents one of the earliest and most widely adopted applications in the medical field. Pioneering work by Lund and Jogestrand in 1997 introduced groundbreaking *in vivo* fluorescence imaging methods for assessing regional cutaneous perfusion in occlusive arterial conditions[45].

Since the dawn of the new millennium, the use of fluorescence imaging techniques has experienced remarkable growth and diversification.

#### Anastomotic perfusion

The use of ICG fluorescence for assessing anastomotic perfusion has become a standard practice in colorectal and oesophageal surgeries, and it facilitates further resection to viable tissue.^[^46-49^]^

Despite advancements in surgical techniques, the morbidity rate after pancreatectomy remains high, ranging from 30% to 50%. Postoperative pancreatic fistula (POPF) is a significant concern; it has a prevalence of 9–15% and is responsible for 33–88% of postoperative deaths[50].

Insufficient vascular perfusion leading to tissue hypoxia is a factor contributing to fistula development, which affects both pancreatic anastomosis and the remaining pancreas^[^51,52^]^.

Traditionally, the evaluation of pancreatic and anastomotic perfusion in clinical settings rely on subjective observations, such as tissue colour, pulsation, and bleeding at the surgical cutting edge. Strasberg *et al*’s 2002 study elucidated the intricate vascular dynamics of the pancreatic neck – which is a crucial area in procedures such as PD – and emphasized its complex arterial supply that originates from branches of the gastroduodenal artery, the SMA, and the splenic artery, thus rendering it a vascular watershed. In their pursuit of enhancing the safety of pancreatic reconstruction, they advocated for an anastomotic approach prioritizing the preservation of adequate blood flow to the pancreas. Their protocol included the meticulous evaluation of the blood supply present at the pancreatic resection margin. Moreover, they proposed the performance of further pancreatic resection, if necessary, to relocate the anastomosis away from the vascular watershed zone. A major criterion that they suggested for assessing whether blood supply was sufficient was the cessation of bleeding upon suturing at the pancreatic cut surface[53].

Despite these efforts, subjective assessments have demonstrated limited reliability in predicting anastomotic leakage. Consequently, several studies have explored the potential of employing ICG fluorescence imaging to assess perfusion in the pancreatic remnant and anastomotic sites^[^12,52,54^]^. Unlike doppler ultrasound, ICG fluoroscopy offers a more straightforward learning curve, thereby reducing variability among users. It provides superior selectivity for blood vessels and enables the precise visualization of microvascular perfusion[52].

Variability in the timing and dosage of ICG administration is evident in the literature. Doussot *et al* conducted a study involving 30 patients undergoing PD. After the injection of 0.1 mg/kg ICG, pancreatic stump perfusion was predominantly observed within 30 seconds. Among this group, six patients experienced pancreatic stump hypoperfusion. Notably, one patient exhibited 2.5 cm of hypoperfused pancreas, which necessitated further resection before anastomosis and resulted in an uneventful recovery. However, the remaining five patients demonstrated incomplete perfusion but they did not undergo additional resection; four of these patients consequently developed a postoperative pancreatic fistula[52].

In a case report by Subar *et al*, a dose of 0.5 mg/kg of ICG was administered intravenously just before the pancreatico-jejunostomy was performed. The contrast resulting from ICG unveiled an ischaemic area at the pancreatic margin that indicated the necessity for further resection before the anastomosis was performed. Remarkably, the patient did not encounter any postoperative pancreatic leaks[54].

Similarly, Rho *et al* injected a patient with 5 mg of IV ICG approximately 3 minutes before the anastomosis was performed during a laparoscopic PD. The ICG exhibited a perfusion defect at the anterior suture line of the pancreatico-jejunostomy that prompted the surgeon to reinforce the site using surgical glue to prevent a major leak[12] (Table 3).

**Table 3:** Role of ICG test of anastomotic perfusion

Case/Study	Patient details	Procedure	Use of ICG	Findings	Outcome
Rho SY *et al*[12]	68-year-old female with distal common bile duct cancer	Laparoscopic pylorus-preserving pancreaticoduodenectomy (PD)	ICG imaging system to check perfusion of the anastomosis line post-pancreaticojejunostomy (PJ)	Perfusion defect noted at anterior wall under infrared light, not visible under white light	Developed grade A postoperative pancreatic fistula, discharged without major sequelae
Doussot A *et al*[52].	30 patients with periampullary tumor undergoing pancreatoduodenectomy	Pancreatojejunostomy and pancreas stump (PS) perfusion assessment using intraoperative fluorescence angiography (IOFA) (January 2020—November 2020)	IOFA of pancreas stump before pancreatojejunostomy	6 patients (20%) had PS hypoperfusion; significant association with occurrence of Postoperative acute pancreatitis (POAP) and CT-confirmed POAP	Clinically relevant POPF rate was 40% with PS hypoperfusion vs. 4% with normal PS perfusion; PS IOFA considered safe and reliable for assessing surgical risk
Subar D *et al*[54]	39-year-old female with invasive ampullary adenocarcinoma	Laparoscopic-assisted pancreaticoduodenectomy (Whipple’s procedure)	Infrared scanning of injected ICG to assess viability of remnant pancreas margin before pancreaticojejunal anastomosis	Ischemic segment identified and resected	No postoperative evidence of pancreatic leak, discharged on postoperative day 18

#### Remnant pancreas and spleen perfusion

The laparoscopic Warshaw technique is an organ-sparing approach for treating benign and low-grade malignant tumours that are situated in the body–tail region of the pancreas. However, postoperative complications such as infection and abscess formation may arise due to compromised blood flow to the spleen[55]. Ebihara *et al* introduced laparoscopic real-time vessel navigation with ICG fluorescence during performance of the laparoscopic Warshaw technique. Using NIR fluorescence, they successfully identified the left gastroepiploic artery and preserved connecting tissue, including vessels around the splenic hilum. Postoperative CT scans revealed no complications related to splenic ischemia or varices formation in their initial experience with three patients[55].

In a recent study, Takagi *et al* demonstrated the assessment of splenic perfusion using ICG fluorescence during a robot-assisted spleen-preserving distal pancreatectomy. They initially simulated the Warshaw technique and assessed the splenic perfusion using ICG after clamping the splenic artery through the gastrosplenic ligament. Subsequently, they performed spleen-preserving distal pancreatectomy using the Kimura technique and evaluated splenic perfusion using ICG[56].

ICG fluorescence plays a crucial role also in pancreas-sparing surgeries for patients with multiple pancreatic tumors. Total pancreatectomy is associated with adverse consequences such as insulin-dependent diabetes, weight loss, and severe diarrhoea with malabsorption[57].

Miura *et al* proposed middle-segment-preserving pancreatectomy (MSPP) as an alternative to total pancreatectomy, and they emphasized the importance of perfusion of the remnant pancreas parenchyma[58].

In a recent study, Iguchi *et al* utilized ICG fluorescence to confirm perfusion to the pancreatic remnant during a MSPP performed in a patient with pancreatic cancer of the uncus and IPMN of the tail[59]. Preservation of the feeding artery into the pancreas, particularly the dorsal pancreatic artery (DPA), is essential to prevent postoperative pancreatic infarction after MSPP.^[^58,60-62^]^ Iguchi *et al* successfully performed MSPP while preserving the DPA—in which they confirming perfusion of the pancreatic remnant perfusion through the IV injection of 10 mg (0.2 mg/kg) ICG—without subsequent symptomatic hypoglycemia, steatorrhea, or malabsorption[59].

Umemoto *et al* recently performed a PD while preserving the pancreatic body in a patient with head of pancreas cancer who had previously undergone total gastrectomy and distal pancreatectomy. They used an IV injection of 7.5 mg ICG to assess the perfusion of the pancreatic remnant postresection, thereby preserving pancreatic endocrine function[63].

Moreover, the utilization of ICG has extended to assessing perfusion in the remnant pancreas after the performance of a pancreatic necrosectomy for acute pancreatitis. A recent case report demonstrates its efficacy in enabling necrosectomy under direct vision, facilitating the procedure until viable pancreatic tissue is identified through ICG fluorescence[64] (Table 4).

**Table 4:** Remnant pancreas and spleen perfusion

Case/Study	Patient details	Procedure	Use of ICG	Findings	Outcome
Takagi K. *et al*[56]	50-year-old female with mucinous cystic neoplasm	Robotic spleen-preserving distal pancreatectomy (SPDP)	ICG fluorescence to check splenic perfusion with and without splenic artery clamping	Good splenic perfusion through gastrosplenic ligament (Warshaw) and splenic artery (Kimura)	Uneventful postoperative course, no complications
Iguchi T. *et al*[59]	79-year-old male with diabetes and pancreatic tumor	Middle segment-preserving pancreatectomy (MSPP)	ICG fluorescence to confirm perfusion to pancreatic remnant	Confirmed perfusion to 4.6 cm pancreatic remnant	Developed grade B pancreatic fistula, managed conservatively; insulin required but no exocrine dysfunction with enzyme supplementation
Umemoto, K. *et al*[63]	66-year-old female with pancreatic head cancer and previous total gastrectomy and distal pancreatectomy	Central pancreatic body-preserving pancreatoduodenectomy	Preoperative angiography and intraoperative ICG fluorescence to evaluate vascular supply	Accurate resection preserving blood flow to pancreatic body	Preserved pancreatic endocrine function, successful R0 resection

#### Perfusion of the remnant stomach during distal pancreatectomy

In situations necessitating concurrent distal pancreatectomy and distal gastrectomy, the preservation of a left inferior phrenic artery (LIPA) is paramount to ensure adequate blood flow to the remnant stomach. Asari *et al* demonstrated the utility of ICG in verifying the presence of LIPA and assessing the perfusion of the remnant stomach in a patient with a distal pancreatic tumor who had previously undergone distal gastrectomy[65]. However, some patients may lack sufficient collateral blood flow to the remnant stomach from the LIPA for such a procedure to be used, meaning that a shift in surgical strategy is required.

Morimoto *et al* employed ICG to evaluate the perfusion of the gastric remnant stomach in a case involving distal pancreatectomy and gastrectomy. The fluorescence pattern revealed limited perfusion (only 3 cm distal from the cardia) that indicated the absence of LIPA, and a total gastrectomy was performed to avoid the anastomotic leak[66].

Kawaguchi *et al* reported a successful case of simultaneous laparoscopic distal gastrectomy and spleen-preserving distal pancreatectomy. They preserved the splenic artery and vein – as well as the gastro-splenic ligament – to prevent stomach ischemia by visualizing the blood supply using ICG fluorescence angiography. The injection of 7.5 mg of ICG enabled the assessment of blood flow to the remnant stomach using a 1588 AIM camera system with endoscopic near-infrared visualization[67] (Table 5).

**Table 5:** Perfusion of the remnant stomach during distal pancreatectomy

Case/Study	Patient details	Procedure	Use of ICG	Findings	Outcome
Asari, S. *et al*[65]	68-year-old man with previous distal gastrectomy for gastric cancer	Distal pancreatectomy (DP)	ICG fluorography and digital subtraction angiography (DSA) of remnant stomach vessels	ICG: Gradual fluorescence of remnant stomach, intensified over entire area; DSA: left inferior phrenic artery (LIPA) distributed to more than half of remnant stomach	Useful for confirming blood flow; LIPA critical for remnant stomach blood supply in absence of left gastric and splenic arteries
Morimoto, M. *et al*[66]	80-year-old man with concurrent pancreatic and gastric cancer	Distal pancreatectomy and distal gastrectomy	ICG evaluation of remnant stomach	Fluorescence extending only 3 cm distal from the cardia	Total gastrectomy performed to avoid ischemic changes and potential complications
Kawaguchi, S. *et al*[67]	55-year-old man with early stomach cancer and intraductal papillary mucinous neoplasms of pancreatic tail	Simultaneous laparoscopic distal gastrectomy (LDG) and spleen-preserving distal pancreatectomy (LSPDP)	ICG fluorescence angiography to visualize blood flow to remnant stomach	Sufficient blood supply to remnant stomach confirmed	No perioperative remnant stomach ischemia, successful Roux-en-Y reconstruction

#### Duodenum and bile duct perfusion

DPPHR has emerged as a viable treatment option for benign and low-grade malignant pancreatic tumors, particularly in younger patients with extended life expectancies such as PanNETs. DPPHR is technically demanding and time-consuming compared with PD, which is primarily due to the need to preserve the blood supply to the common bile duct (CBD) and the duodenum. Laparoscopic techniques offer distinct advantages over open surgery, such as providing enhanced visualization of vascular arcades and branches – which is crucial for their preservation – owing to the expanded surgical field.

In a case series involving 22 patients undergoing laparoscopic DPPHR, Hong *et al* utilized ICG injection to identify and safeguard the vascular supply of the CBD and the ampulla of Vater along with the posterior superior pancreaticoduodenal artery and its branches. ICG fluorescence facilitated the confirmation of CBD and duodenal perfusion, as well as the detection of postoperative bile leaks[68].

Similarly, Huang *et al*, in a related series, showed the usefulness of ICG in guiding surgeons to delineate the vascular anatomy surrounding the duodenum and the CBD, thus aiding in the preservation of the posterior pancreaticoduodenal arch, which is crucial for duodenal and CBD perfusion within the pancreatic segment[41].

The evaluation of duodenal perfusion is extremely important in pancreas transplantation, in which avoiding duodenal ischemia can be of the utmost importance for the survival of the transplanted specimen. Recent case series demonstrate the efficacy of ICG in assessing duodenal wall perfusion relative to recipient tissue perfusion^[^69,70^]^. Panaro *et al* administered 0.5 mg/kg ICG after performing a vascular anastomosis, which enabled visualization of the arterial vascularization of the graft duodenal stump within 35 seconds. The suspicion of ischemia prompted the performance of a duodenal stump resection in one case. Additionally, ICG-facilitated the assessment of perfusion at the pancreas and the site of duodeno-jejunal anastomosis[70] (Table 6).

**Table 6:** Duodenum and bile duct perfusion

Case/Study	Patient Details	Procedure	Use of ICG	Findings	Outcome
Huang, J. *et al*[42]	30 patients (11 males, 19 females, age 19-76)	Laparoscopic Duodenum-preserving pancreatic head resection (LDPPHR)	ICG vs. non-ICG groups	Lower intraoperative bile duct injury (10% vs 60%); shorter operation time in ICG group	Reduced bile duct injuries and postoperative complications with ICG
Hong D. *et al*[68]	22 patients undergoing LDPPHR	Laparoscopic duodenum-preserving pancreatic head resection (LDPPHR)	ICG to visualize and preserve CBD and duodenum vessels	Shorter pancreatic duct end-to-end anastomosis time than PJ; comparable	Safe and efficient; all complications resolved conservatively
Garcia-Roca, R. *et al*[69]	General cases in pancreas transplantation	Pancreas transplantation	ICG to evaluate duodenal stump perfusion	Perfusion assessment within seconds, enabling real-time surgical decisions	Uneventful perioperative period; no biliary or anastomotic complications
Panaro, F *et al*[70]	6 cirrhotic patients and 5 kidney-pancreas transplant patients	Liver and kidney-pancreas transplantations	ICG to assess biliary duct and duodenal stump perfusion	No biliary leaks or stenosis after liver transplant; no anastomotic complications in pancreas transplant	Provided important perfusion information; potentially reduced anastomotic leakage risks

#### Vascular anastomosis perfusion

Advancements in perioperative chemotherapy and radiation therapy have broadened the scope for curative resection in pancreatic cancer, even extending to patients who were initially deemed unsuitable for resection to treat locally advanced PDAC[71]. Within this context, complex procedures such as pancreatectomy involving arterial resection and/or reconstruction are often necessary to achieve R0 resection, particularly for tumors located in the pancreatic neck and body. Despite their significantly high postoperative morbidity rates, procedures such as pancreatoduodenectomy with common hepatic artery resection (PD-CHAR), distal pancreatectomy with celiac axis resection (DP-CAR), and pancreatoduodenectomy with splenic artery resection (PD-SAR) have become increasingly common in specialized HPB centres.^[^72-75^]^

A crucial aspect revolves around surgeons’ abilities to intraoperatively confirm blood flow through reconstructed arteries or into organs that are supplied by the remaining blood vessels. In this context, ICG fluoroscopy has emerged as a valuable tool that enables the navigation of surgery around vessels and the assessment of organ perfusion postresection. The intraoperative detection of technical errors can pre-empt the need for re-exploration surgery, thereby averting complications such as anastomotic occlusion or leakage. In a prospective pilot study involving nine patients, Okada *et al* showcased the efficacy of ICG in assessing blood flow in patients undergoing PD-CHAR after neoadjuvant chemotherapy for neck/body pancreatic cancer. After the injection of 2.5 mg/ml of ICG, infrared observation via the photodynamic eye (PDE) system confirmed the presence of blood flow in various critical sites, including the arterial anastomosis between the transected proper hepatic artery and the splenic artery, the portal vein anastomosis, and vital organs such as the liver, the stomach, and the spleen[76].

In cases of celiac axis invasion, the performance of DP-CAR has become increasingly common in certain institutions and regions, despite its associated high-morbidity rates, even in high-volume centres. Nakamura *et al*, in a study involving 80 patients, reported postoperative rates of 28.8% for ischaemic gastropathy and 25% for delayed gastric emptying (DGE)[77]. When the celiac artery is excised, preserving and confirming the arterial arcade that is responsible for the perfusion of the liver and the residual stomach via collateral circulation becomes imperative. The necessity of preoperative hepatic artery embolization remains controversial, with many centres opting against it due to the persistent occurrence of ischaemic gastropathy. Notably, Okada *et al* found that preserving the left gastric artery (LGA) significantly reduced the incidence of DGE[73].

In an effort to mitigate the risk of ischaemic gastropathy or DGE post-resection of the LGA, Sato *et al* proposed LGA reconstruction using the middle colic artery or the jejunal artery in a preliminary study in 2016[78]. Subsequently, in a series of 18 patients, they utilized ICG fluorescence imaging to assess vascular patency, which aided in the identification of patients requiring re-anastomosis. Among the 18 patients, 5 patients required re-anastomosis, good flow was demonstrated in 14 patients, and poor flow was observed in 4 patients. DGE occurred in 14% of patients with good flow and 75% of patients with poor flow, and ischaemic gastropathy occurred in two patients who had poor flow[27].

Furthermore, ICG fluorescence serves as an invaluable tool for assessing vascular supply to the stomach and liver, and for facilitating the laparoscopic approach to DP-CAR[79].

## Limitations

Narrative reviews are prone to several limitations as they rely heavily on the authors’ selection of reports from the literature and interpretations of findings. Firstly, the interpretation of findings in a narrative review is subjective, which can affect the overall reliability and validity of the review; different reviewers can come to different conclusions based on their individual biases and perspectives.

Secondly, unlike systematic reviews, narrative reviews do not follow a strict protocol for conducting literature searches and selecting publications. This may result in authors overlooking relevant studies or failing to consider all available evidence. Thirdly, this review may lack generalizability due to the heterogeneous nature of the included studies and the absence of standardized data extraction methods. As a result, the conclusions drawn may not apply universally to all populations or settings. Finally, this narrative review does not include statistical analysis or meta-analysis, which may limit the strength of the conclusions that have been drawn.

## Conclusion

The role of ICG in pancreatic surgery is far from defined; as reported in this narrative review, its applications and benefits can be manifold. However, for each of the purposes proposed in this review and in clinical practice, there are no standardized methodological or dosing protocols for ICG that can ensure reproducibility of the best possible outcome. Future studies should aim to standardize the methodology for each specific application to optimize results and should use IGT only when it is truly indicated.

## Data Availability

Available upon reasonable request.
